# Efficacy Evaluation of “Enhanced” Natural Killers with *CISH* and *B2M* Knockouts on Viability and Metabolic Status of 3D Glioblastoma Spheroid Cells in Patients

**DOI:** 10.17691/stm2025.17.1.10

**Published:** 2025-02-28

**Authors:** D.V. Yuzhakova, D.A. Sachkova, M.V. Shirmanova, V.I. Shcheslavskiy, A.M. Mozherov, E.B. Dashinimaev, V.P. Baklaushev, G.M. Yusubalieva

**Affiliations:** PhD, Senior Researcher, Laboratory of Genomics of Adaptive Antitumor Immunity, Research Institute of Experimental Oncology and Biomedical Technologies; Privolzhsky Research Medical University, 10/1 Minin and Pozharsky Square, Nizhny Novgorod, 603005, Russia; Senior Researcher, Laboratory of Cellular Technologies; Federal Scientific and Clinical Center of the Federal Medical Biological Agency of Russia, 28 Orekhovy Blvd., Moscow, 115682, Russia; Laboratory Assistant, Laboratory of Fluorescent Bioimaging, Research Institute of Experimental Oncology and Biomedical Technologies; Privolzhsky Research Medical University, 10/1 Minin and Pozharsky Square, Nizhny Novgorod, 603005, Russia; PhD Student, Department of Biophysics, Institute of Biology and Biomedicine; National Research Lobachevsky State University of Nizhny Novgorod, 23 Prospekt Gagarina, Nizhny Novgorod, 603022, Russia; PhD, Deputy Director for Science, Research Institute of Experimental Oncology and Biomedical Technologies; Privolzhsky Research Medical University, 10/1 Minin and Pozharsky Square, Nizhny Novgorod, 603005, Russia; DSc, Head of the Laboratory of Optical Spectroscopy and Microscopy, Research Institute of Experimental Oncology and Biomedical Technologies; Privolzhsky Research Medical University, 10/1 Minin and Pozharsky Square, Nizhny Novgorod, 603005, Russia; Junior Researcher, Laboratory of Optical Spectroscopy and Microscopy, Research Institute of Experimental Oncology and Biomedical Technologies; Privolzhsky Research Medical University, 10/1 Minin and Pozharsky Square, Nizhny Novgorod, 603005, Russia; PhD, Acting Head of the Laboratory of Cellular Reprogramming, Research Institute of Translational Medicine; Pirogov Russian National Research Medical University, 1, Bldg. 6, Ostrovityanova St., Moscow, 117513, Russia; Head of the Laboratory of Bioengineering, Research Institute of Molecular and Cellular Medicine; Peoples’ Friendship University of Russia named after Patrice Lumumba, 6 Miklukho-Maklaya St., Moscow, 117198, Russia; MD, DSc, Head of the Biomedical Research Center; Federal Scientific and Clinical Center of the Federal Medical Biological Agency of Russia, 28 Orekhovy Blvd., Moscow, 115682, Russia; Head of the Cell Therapy Development Department; Federal Center of Brain Research and Neurotechnologies of the Federal Medical Biological Agency of Russia, 1, Bldg. 10, Ostrovityanova St., Moscow, 117513, Russia; Head of the Laboratory of Molecular Regeneration Mechanisms; Engelhardt Institute of Molecular Biology of the Russian Academy of Sciences, 32 Vavilov St., Moscow, 119991, Russia; MD, PhD, Senior Researcher, Laboratory of Cell Technologies; Federal Scientific and Clinical Center of the Federal Medical Biological Agency of Russia, 28 Orekhovy Blvd., Moscow, 115682, Russia; Head of the Laboratory of Solid Tumor Immunotherapy; Federal Center of Brain Research and Neurotechnologies of the Federal Medical Biological Agency of Russia, 1, Bldg. 10, Ostrovityanova St., Moscow, 117513, Russia; Senior Researcher, Laboratory of Molecular Regeneration Mechanisms; Engelhardt Institute of Molecular Biology of the Russian Academy of Sciences, 32 Vavilov St., Moscow, 119991, Russia

**Keywords:** immunotherapy, NK cells, FLIM, metabolic imaging, tumor spheroids, patient’s glioblastoma, NAD(P)H

## Abstract

One of the alternative approaches to glioblastoma treatment is cellular immunotherapy based on natural killer cells (NK cells). To enhance their cytotoxic effect on tumor cells, new NK cell lines are being created using genetic engineering techniques.

**The aim of the study** was to evaluate the impact efficacy of “enhanced” NK cells on early metabolic rearrangements and the viability of glioblastoma cells in a patient using a tumor spheroid model.

**Materials and Methods:**

The study used a primary culture of GBM7-Luc2-mKate2 human glioblastoma, a line of YT (YTwt) wildtype human NK cells, as well as lines created by us with overexpression of VAV1 protein with either *CISH* (YT–Vav1^+^CISH^–/–^) or *B2M* (YT–Vav1^+^B2M^–/–^) knockouts. Tumor spheroids were produced in round-bottomed, low-adhesive plates. 100 thousand immune cells were added to each spheroid, and spheroids viability was evaluated at several time points applying fluorescence staining using a live/dead cell viability assay kit; autofluorescence of metabolic coenzyme nicotinamide adenine dinucleotide (phosphate), or NAD(P)H, was visualized in spheroids using an LSM 880 laser scanning microscope (Carl Zeiss, Germany) with a FLIM module (Becker & Hickl GmbH, Germany).

**Results:**

It was found that autofluorescence attenuation parameters of NAD(P)H coenzyme in human glioblastoma cells change significantly when exposed to both YT–Vav1^+^CISH^–/–^ and YT–Vav1^+^B2M^–/–^, indicating occurrence of an early metabolic shift in tumor cells towards a less aggressive oxidative phenotype, and this is consistent with dead cells fraction increase and living cells fraction decrease in spheroid composition.

**Conclusion:**

The data obtained on enhanced cytotoxic activity of new modified NK cell lines against human glioblastoma spheroids are important to understand interaction mechanisms between tumor and immune cells and the development of glioblastoma adoptive cell therapy.

## Introduction

Among the existing approaches to malignant tumors treatment, one of the promising strategies is immunotherapy aimed at immune system activation against tumor cells [1, 2]. Immunotherapy has already proven its efficacy against a number of oncological diseases, and now scientists are working to develop personalized approaches and expand the range of its use localizations. Of particular interest is the development of glioblastoma immunotherapy, the most aggressive form of brain tumors. Until now, this remains a challenge as blood-brain barrier along with immunosuppressive tumor microenvironment hamper the achievement of a positive antitumor response [3, 4].

The use of natural killer cells (NK cells) is considered a promising area of cellular antitumor immunotherapy. These cells are a component of innate immunity and play a key role in recognizing and destroying cells infected with viruses and tumor cells. NK cells are functionally similar to CD8^+^ T lymphocytes, however, unlike them, they are able to recognize and act against pathological cells without additional sensitization by antigens [5, 6].

The cytotoxic activity of NK cells is controlled by inhibitory and activating receptors on their surface. As a rule, the cytotoxic activity of NK cells entering the tumor is low due to the influence of various immunosuppressive factors and regulatory T cells. To enhance the antitumor activity of NK cells, genetic engineering techniques are used, namely genetic constructs are introduced to increase the signaling proteins expression involved in cell activation and knockout of genes associated with their cytotoxicity inactivation [7–9]. It is assumed that the combination of such NK cell modifications can provide the most evident cytotoxic effect.

Earlier [10], we created two modified cell lines with increased cytotoxicity based on NK-like human YT cells with overexpression of VAV1 protein with *CISH* or *B2M* knockouts. VAV1 protein is a member of the family of guanine nucleotide exchange factors, one of the functions of which is the positive regulation of NK cell-mediated cytotoxicity [11]. The *CISH* gene encodes CIS, a cytokine-inducible SH2-containing protein. This gene produces a negative regulator of NK cells cytotoxicity, reducing their sensitivity to interleukin 15 (IL-15) [12]. The *B2M* gene encodes β2-microglobulin protein, a component of the light chain of the main histocompatibility complex class I (MHC I). *B2M* knockout disrupts MHC I NK cells structure making these cells invisible to host T lymphocytes, thereby opening up prospects for widespread clinical use of allogeneic NK cells [13].

Our previous study [10] demonstrated that modified NK cell lines eliminate primary glioblastoma cells in monolayer culture more efficiently than wild-type cells.

**The aim of the study** was to evaluate the efficacy of “enhanced” NK cells impact on early metabolic rearrangements and the viability of patients’ glioblastoma cells using a three-dimensional model of tumor spheroids.

Tumor spheroids are spherical multicellular aggregates with a heterogeneous structure and thus act as a more complex cellular model compared to standard monolayer cultures. Due to their spatial organization, spheroids allow to model tissue three-dimensional architecture with characteristic intercellular interactions and tight contacts, oxygen and nutrient gradients, variable levels of cells proliferative/metabolic activity and viability. Spheroids and similar multicellular structures (e.g. explants, organoids) obtained from the patients’ tumor materials are recognized as adequate models for preclinical testing and personalized selection of antitumor therapy [14].

Traditionally, cell viability and proliferative activity are the base to assess tumor cell response to therapy at *in vitro* systems using colorimetric methods, fluorescent dyes or immunocytochemistry. Fluorescence lifetime imaging microscopy (FLIM) of coenzyme NAD(P)H autofluorescence is considered a promising method to assess cellular response to therapy [15, 16]. This method allows to assess living cells metabolic status dynamics without additional staining and destruction considering intercellular heterogeneity. The “metabolic” FLIM is based on assessment of NAD(P)H fluorescence (attenuation) lifetime which varies for different coenzyme types — free and protein-bound, unphosphorylated, and phosphorylated. Metabolic perturbations accompany proliferation decrease or precede cell death after exposure and inevitably lead to changes of NAD(P)H fluorescence decay parameters [17, 18].

Thus, the therapeutic efficacy research of “enhanced” NK cells on patient-specific tumor spheroids with early response assessment using FLIM method is an innovative aspect of studying the described problem.

## Materials and Methods

### Cell cultures

A previously obtained primary culture of GBM7-Luc2-mKate2 patient’s glioblastoma was used to create tumor spheroids [19]. To obtain the required amount, tumor cells were cultured in DMEM nutrient medium (PanEco, Russia) according to the protocol we developed earlier [20].

The research was run using a line of NK-like wildtype human cells (YTwt) kindly provided by A.V. Filatov (Immunochemistry Laboratory of Moscow Institute of Immunology, Russia) along with modified lines created on this basis with overexpression of VAV1 protein with either *CISH* (YT–Vav1^+^CISH^–/–^) or *B2M* (YT– Vav1^+^B2M^–/–^) knockouts [10].

NK-like YT cell lines were cultured at the same conditions as tumor cells, but IL-2 (NPC Biotech, Russia) and IL-15 (Sino Biological, China) were always added to the nutrient medium (1 μl per 1 ml of medium). During passaging, the cells were centrifuged for 5 min at 300 g, and the precipitate was resuspended in a nutrient medium.

### Tumor spheroids derivation

To obtain 3D tumor spheroids, 2000 cells along with 200 μl of nutrient medium were seeded to each well of 96-well lowadhesive round-bottomed plates (Corning, USA) [20]. Spheroid morphology was evaluated using a DM IL LED inverted microscope (Leica Microsystems, Germany).

On day 4 of growth, the spheroids were transferred using a pipette onto special plates with black walls and glass bottom (Gibco, USA) — 1 spheroid per well in DMEM nutrient medium without phenol red (Gibco, USA) followed by examination applying fluorescence microscopy.

100 thousand of YTwt, YT–Vav1^+^CISH^–/–^ or YT– Vav1^+^B2M^–/–^ cells were added to each spheroid according to a previously developed protocol [10].

### Assessment of spheroids viability using fluorescent staining

To study the mechanisms of tumor cell death after incubation with NK cells, spheroids were stained using a live/dead cell viability assay kit (ab 176750; Abcam, Great Britain). According to the manufacturer’s protocol, a mixture of solutions was used — Nuclear Green DCS1 to stain necrotic/late apoptotic cells and CytoCalcein Violet 450 to stain viable cells. Fluorescent images of spheroids were obtained using a Leica DM IL LED inverted microscope (Leica Microsystems, Germany) with YFP (Ex: BP 500/20, Em: BP 535/30) filters for necrotic/late apoptotic cells and CFP (Ex: BP 436/20, Em: BP 480/40) filters for viable cells. The stained fraction of viable and necrotic/ late apoptotic cells in the spheroid was calculated as a percentage using the ImageJ program (National Institutes of Health, USA).

### Fluorescence microscopy and FLIM

An LSM 880 laser scanning confocal microscope (Carl Zeiss, Germany) was used during the research. The excitation source was a femtosecond Ti:Sa laser (Spectra Physics, USA) with 80 MHz repetition rate of 120 fs duration pulses and 690 to 1040 nm wavelength tunable range. The images were obtained using a 40×/1.3 oil immersion lens. During experiments, the spheroids were maintained in 5% CO_2_ medium at 37°C. To excite fluorescence of red mKate2 protein in spheroid tumor cells, 543 nm wavelength irradiation was applied, and a 650 nm wavelength signal was received.

Fluorescence lifetimes were detected using a FLIM module based on a time-correlated count of single TCSPC photons (Becker & Hickl, Germany). Coenzyme NAD(P)H fluorescence was called applying two photon 750 nm excitation, the received signal was within 450 to 490 nm wavelength range. The excitation irradiation power was 7 mW. The number of photons per pixel was not less than 5000 at ~60 s photon collection time.

To evaluate fluorescence decay parameters, SPCImage software (Becker & Hickl GmbH, Germany) was used. The decay was approximated applying the least squares method along with a bi-exponential function at 0.8 to 1.2 confidence interval of Chi-square approximation. The following decay parameters were calculated: τ_*1*_ — the lifetime of the short component, τ_*2*_ — the lifetime of the long component, α_*1*_ — the percentage contribution of the short component, α_*2*_ — the percentage contribution of the long component, τ_*m*_ — weighted mean lifetime (τ_*m*_=α_*1*_·τ_*1*_+α_*2*_·τ_*2*_)/(α_*1*_+α_*2*_). Short lifetime τ_*1*_ (0.3 to 0.5 ns) corresponds to free NAD(P)H produced during glycolysis process. Long lifetime τ_*2*_ (1.2 to 2.5 ns) corresponds to protein-bound NAD(P)H, its contribution correlating with mitochondrial respiration activity.

The cytoplasm area of each spheroid cell was examined. Images of 4 to 10 spheroids were obtained for each exposure and control with no exposure. 15 to 20 cells of each spheroid were examined.

### Statistical analysis

GraphPad Prism 8.4.3 software (GraphPad Software, USA) was used for data comparative analysis and graphical representation. Data were checked for distribution normality using Shapiro–Wilk test (p≥0.05 distribution was considered normal). Since Shapiro–Wilk test revealed abnormal distribution, nonparametric Mann–Whitney U test was used to assess statistically significant differences between research groups, and p<0.05 differences were considered statistically significant.

## Results

### Assessment of human glioblastoma tumor spheroids viability after exposure to “enhanced” NK cells

Cytotoxicity of “enhanced” NK cells against human glioblastoma tumor spheroids was assessed evaluating spheroid morphological state according to data of light microscopy and fluorescence staining using a live/dead cell viability assay kit 3 and 24 h after immune cells were added.

**Figure 1.**
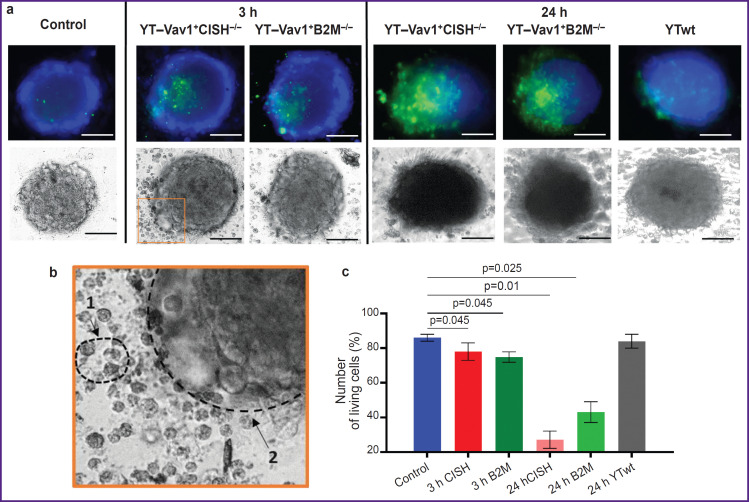
Viability assessment of tumor cells in spheroids via fluorescence staining of live/dead cells after incubation with NK cell lines: (a) microscopic images of spheroids; superimposition of blue (fraction of living cells) and green (fractions of necrotic and late apoptotic cells) channels and light microscopy images; (b) enlarged image showing NK cells (*1*) and spheroid border (*2*); (c) evaluation of living cells fraction in spheroids after 3 and 24 h of exposure. Columns represent percentage values of living cells fraction in spheroids, vertical lines represent the standard error of the mean (n=5 to 8 spheroids); bar — 180 μm

The control group spheroids (with no immune cells added) shape was spherical, their structure was dense with a clear and even border. They consisted mainly of living cell fraction (~86%). Necrotic cells accounted for ~14% and were observed only at the spheroid central zone (Figure 1).

Spheroid morphology showed no visible changes when spheroids where incubated with unmodified YTwt NK cell line for 24 h. Spheroids retained their dense structure, and a small number of detached cells was observed at spheroid periphery. No difference in viable cell fraction was found compared to the intact control (~84%).

After 3 h of incubation with modified YT–Vav1^+^CISH^–/–^ and YT–Vav1^+^B2M^–/–^ NK cell lines, the spheroids lost their compact structure and tumor cells detached along the edge. Fluorescence microscopy showed that spheroid dead cell fraction increased (~21% for YT–Vav1^+^CISH^–/–^, ~25% for YT–Vav1^+^B2M^–/–^) and, accordingly, living cell fraction decreased (~78% for YT–Vav1^+^CISH^–/–^, ~74% for YT–Vav1^+^B2M^–/–^; p=0.045 compared with the control). At light microscopy images, a tumor spheroid surrounds an NK cell population with some cells at spheroid periphery. NK cells morphology was typical — rounded cells with evident granular cytoplasm.

24 h after exposure was started, the spheroids showed signs of destruction. At spheroid border, evident detachment of cells at the points of contact with immune cells was observed, the spheroid became more elongated and less dense due to intercellular contacts loss. The fraction of dead tumor cells increased to ~71% for YT–Vav1^+^CISH^–/–^ and to ~58% for YT–Vav1^+^B2M^–/–^ (p=0.01 and p=0.025, respectively, compared to the control with no exposure).

Thus, the viability assessment of human glioblastoma spheroids after exposure to modified NK cell lines demonstrated enhanced cytotoxicity of YT-Vav1^+^CISH^–/–^ and YT–Vav1^+^B2M^–/–^ cells compared with standard unmodified YTwt line, this being consistent with earlier experiments on a human glioblastoma monolayer culture of [10].

### Assessment of tumor spheroid cell metabolic status after exposure to “enhanced” NK cells using FLIM microscopy

**Figure 2.**
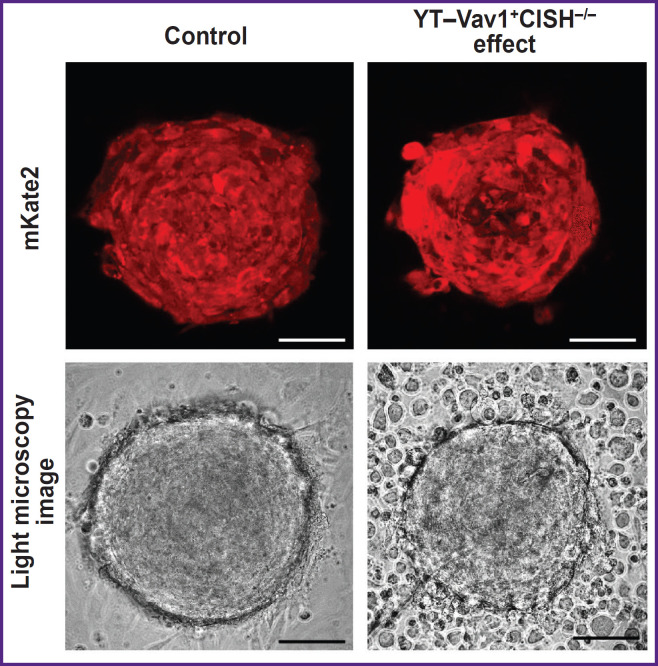
Confocal fluorescence microscopy of 3D human glioblastoma spheroids during incubation with YT–Vav1^+^CISH^–/–^ NK cell lines Representative fluorescent images in red fluorescent protein mKate2 channel of spheroid tumor cells; bar — 100 μm

FLIM microscopy was used to obtain images of NAD(P)H coenzyme autofluorescence in spheroid cells of patient’s glioblastoma and to assess lifetime parameters. To study the effect of NK cells on tumor cell metabolism, it was assessed 6 h after incubation with wild-type YTwt line and 2, 4, and 6 h after YT– Vav1^+^CISH^–/–^ or YT–Vav1^+^B2M^–/–^ knockout lines were added. To identify spheroid tumor cells, fluorescent confocal images of spheroids were obtained without and with addition of NK cell lines to the fluorescence channel of red protein mKate2 (Figure 2).

### Effects of YTwt cells on tumor spheroids

**Figure 3.**
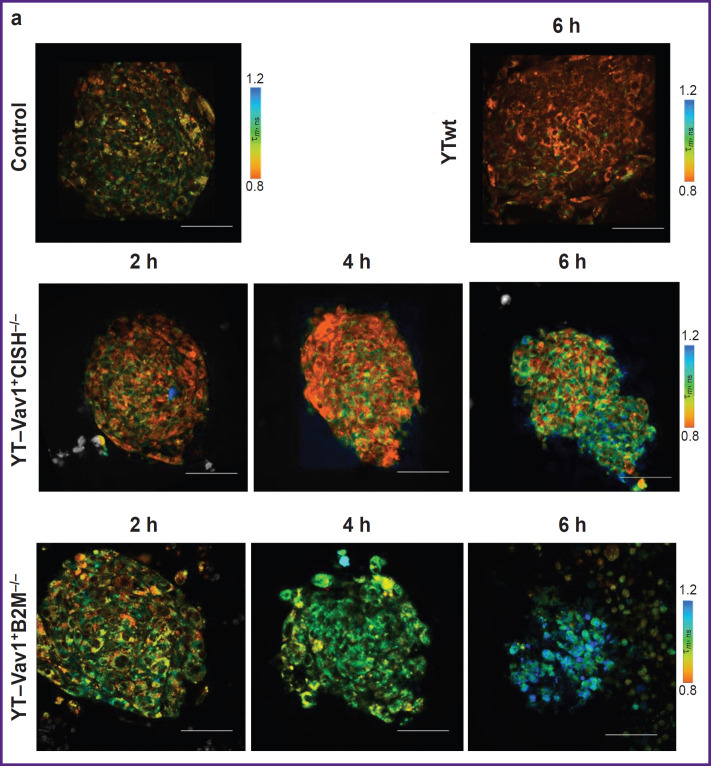

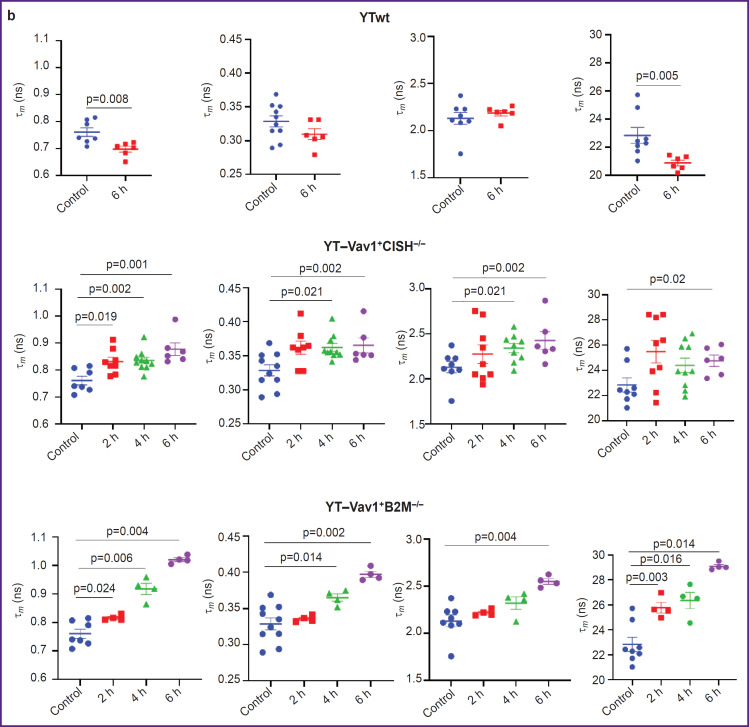
FLIM microscopy of 3D human glioblastoma spheroids after incubation with standard and “enhanced” NK cell lines: (a) representative FLIM images of spheroids according to τ_*m*_ parameter in NAD(P)H channel; (b) quantitative analysis of the fluorescence lifetime parameters τ*_m_*, τ_*1*_, τ_*2*_ and α*_2_*. Dots show parameter values of individual spheroids; horizontal lines represent group parameter average value, vertical lines represent the standard error of the mean (n=4 to 10 spheroids each containing 15 to 20 cells). Bar — 100 μm. *Continuation on the next page*

In the control (with spheroid no exposure to immune cells), typical NAD(P)H fluorescence lifetime values were noted: τ_*1*_ ~0.33 ns for short component, τ_*2*_ ~2.1 ns for long component, ~77 and 22% short and long component contribution α_*1*_ and α_*2*_, respectively, ~0.76 ns τ_*m*_ average lifetime. After 6 h of incubation with unmodified YTwt NK cells, a statistically significant decrease to 0.7±0.01 ns (p=0.008) was observed of τ_*m*_ parameter resulting from decrease to 20.9±0.2% (p=0.005) of α_*2*_ bound NAD(P)H relative contribution (Figure 3). Reduced bound NAD(P)H contribution may indicate tumor cell increased glycolysis, in its turn associated with retention and enhancement of glioblastoma cells aggressive phenotype despite the therapeutic effect.

### Effects of YT–Vav1^+^CISH^–/–^ and YT–Vav1^+^B2M^–/–^ NK cells on tumor spheroids

Evident changes in all lifetime parameters were detected in tumor spheroids exposed to YT–Vav1^+^CISH^–/–^ knockout NK cells. After 2 h of incubation, the values of parameters increased: τ_*1*_ from 0.33±0.01 to 0.36±0.01 ns (p=0.019); τ_*2*_ from 2.1±0.6 to 2.2±0.9 ns; α_*2*_ from 22.8±0.10 to 25.9±0.9%; τ_*m*_ from 0.76±0.02 to 0.83±0.20 ns (p=0.019). At the 4^th^ hour of incubation, τ_*1*_ and τ_*m*_ values remained at the same level, changes of τ_*2*_ became statistically significant (2.3±0.5 ns; p=0.021). At the 6^th^ hour, increase was observed: τ_*1*_ to 0.37±0.10 ns (p=0.017), τ_*2*_ to 2.5±0.9 ns (p=0.02), α_*2*_ to 24.2±0.5%, this resulting in τ_*m*_ average lifetime increase to 0.88±0.20 s (p=0.001 compared to control with no exposure).

Incubation of human glioblastoma spheroids with YTVav1^+^ B2M^–/–^ knockout NK cells also showed a 6-hour gradual increase of all fluorescence lifetime parameters. After 2 h of incubation, significant changes occur of α_*2*_ parameter with statistically significant increase of values from 22.8±0.1% to 25.8±0.4% (p=0.03), this resulting in τ_*m*_ average lifetime increase from 0.76±0.02 to 0.82±0.04 ns (p=0.024). At the 4^th^ hour of exposure, a statistically significant increase of τ_*1*_ was detected — from 0.33±0.01 to 0.36±0.03 ns (p=0.014) compared to control spheroids. After 6 h of spheroid incubation with immune cells, all lifetime parameters showed higher values compared to intact control; higher 1.02±0.06 ns (p=0.004) τ_*m*_ values reflected this. It should be noted that effect of YT–Vav1^+^B2M^–/–^ cells caused lower spread of values within spheroid groups for each time point compared to effect of YT–Vav1^+^CISH^–/–^ cells; this indicates greater reproducibility and stability of metabolic dysfunction.

Thus, the effect of modified NK cells on human glioblastoma spheroids has evident cytotoxic impact combined with metabolic rearrangements at cellular level that could be detected applying FLIM method to NAD(P)H coenzyme.

## Discussion

Immunotherapy demonstrates high potential as a treatment method of glioblastoma. Adoptive cell therapy is actively developed — immune cells are obtained from a patient, cultured *in vitro* to increase their number, further modified, and are injected into the body to destroy the tumor. NK cells look promising as an antitumor agent for adoptive cell therapy due to their ability to recognize and destroy malignant cells without requiring antigen-specific activation. However, despite their high therapeutic potential, native NK cells efficacy is low due to the immunosuppressive microenvironment of tumors; therefore, methods are currently being actively developed to “enhance” NK cells cytotoxicity and overcome the mechanisms of immune surveillance evasion. Various cell modification techniques are used for this purpose, e.g. creation of chimeric antigenic receptors or specific knockout genes responsible for reducing NK cells cytotoxicity [21]. Currently, preclinical studies have demonstrated the high efficacy of NK therapy using “enhanced” cells for various types of oncological diseases, including leukemia, lymphoma, myeloma, ovarian cancer [22]. At the same time, treatment of solid tumors using NK cells, in particular glioblastoma [23], causes certain difficulties; therefore, creation of new effective NK cell lines and studies of their cytotoxicity remain urgent research areas.

The most widely used cytokine for NK cell therapy in clinical trials is IL-15. Besides, IL-15 receptor is expressed on both NK cells and CD8^+^ T cells, but, importantly, not on regulatory T cells [24]. The latter provides a key advantage of their use compared to IL- 2-based therapy [25]. Increasing number of publications evidences that exogenous administration of IL-15 prevents loss of NK cell effector functions in tumor microenvironment.

However, continuous treatment using human NK cells via IL-15 can cause depletion of these cells, so both the treatment regimen and the administration method to achieve maximum benefit for patients should be carefully considered [26].

Previously [10], we were able to obtain *CISH* knockout lines allowing to increase NK cells cytotoxicity without adding exogenous IL-15 to the culture. Experiments showed that NK cells with *CISH* knockout had a more effective impact on the monolayer of human primary glioblastoma culture.

In addition, the method of metabolic fluorescent timeresolved imaging allowed to obtain valuable data on tumor metabolism features from the model of human glioblastoma 3D spheroids [20]. Combined results allowed to evaluate the efficacy of YT-Vav1^+^CISH^–/–^ and YT–Vav1^+^B2M^–/–^ modified NK cell lines at conditions close to tumor process development in the human body.

In this research, antitumor activity of YT-Vav1^+^CISH^–/–^ and YT–Vav1^+^B2M^–/–^ modified NK cell lines was evaluated using a model of 3D tumor spheroids of a patient’s glioblastoma. The focus of the research was on the study of early metabolic rearrangements in tumor cells during NK therapy using the FLIM optical method. At the moment, metabolic aspects of NK therapy are poorly understood. The advantage of FLIM is the possibility to perform the dynamic observations of living cells with (sub)cellular resolution compared to biochemical, immunocytochemical, and molecular genetic methods of cellular metabolic status assessment.

It was found that all autofluorescence decay parameters of NAD(P)H coenzyme in human glioblastoma cells significantly change when exposed to YT-Vav1^+^CISH^–/–^ and YT–Vav1^+^B2M^–/–^ modified NK cells; this indicates that tumor cell metabolic status is changed and is consistent with data on spheroid dead cells fraction increase and living cells fraction decrease and, accordingly, on effective response to therapeutic effects. In a typical case, contribution growth of bound α_*2*_ NAD(P)H in cells is associated with increased mitochondrial respiration. In one of our studies [20] using FLIM method, we showed that relative contributions of α_*1*_ and α_*2*_ NAD(P)H adequately represent metabolic rearrangements in spheroids of primary glioblastoma in patients as response to oxygen content decrease in their microenvironment. During research [18], we found that α_*2*_ NAD(P)H is strongly correlated to survival as well as to proliferative activity of tumor cells isolated from gliomas of various patients treated with temozolomide. α_*2*_ NAD(P)H contribution increase in tumor cells as a result of switching to a more oxidative metabolism is a typical reaction to various types of cytotoxic chemotherapy shown *in vitro* and *in vivo* at numerous researches of our group and of other laboratories [27–31].

In addition to changes of α_*2*_ bound NAD(P)H relative contribution, NK cells effects on tumor cells also caused absolute lifetime values increase of τ_*1*_ free and τ_*2*_ proteinbound NAD(P)H fluorescence. A number of factors like pH, temperature, and viscosity of local microenvironment affect fluorescence lifetime of various fluorophores, but these factors influence within physiological values range is insignificant for NAD(P)H. A recent study of Song et al. [32] showed that τ_*1*_ free NAD(P)H lifetime depends on the size of oxidized (NAD^+^) total pool and reduced τ_*1*_ coenzyme (NADH) increases with pool decrease and decreases with pool increase. Lifetime of τ_*2*_ enzymebound NAD(P)H depends on specific enzyme availability in a particular metabolic pathway [33].

Activity of many key enzymes of glucose metabolic pathways, including NAD(P)H-binding proteins, is altered during glioblastoma development [34]. Recent researches using FLIM method indicate a relationship of τ_*2*_ NAD(P)H to mitochondrial cell activity. Morrow et al. [35] demonstrated that τ_*2*_ NAD(P)H increases significantly during cell aging. Sanchez et al. research [36] found evident decrease of τ_*1*_ and τ_*2*_ in severe mitochondrial dysfunction. It was shown that increased mitochondrial activity is one of the signs of tumor oxidative phenotype. This suggests that τ_*2*_ NAD(P)H growth when “enhanced” NK cells produce their effect is also associated with a metabolic shift towards oxidative phosphorylation. Obtained data are consistent with our previous research [18] on temozolomide effect against patient’s glioblastoma cells, where we also observed an increase of τ_*1*_ and τ_*2*_ NAD(P)H at therapeutic effect.

Metabolic reprogramming, namely glycolysis activation, is an important biological feature of glioblastoma. Active aerobic glycolysis is used not only for accelerated production of ATP molecules, but also for metabolic intermediates synthesis necessary to maintain rapid growth and proliferation of glioblastoma. Reprogramming of cellular metabolism is closely related to changes of signaling pathways, in particular PI3KAkt- mTOR; its activation in glioblastoma cells enhances glucose molecules absorption by cells and alters the activity of certain enzymes involved in glycolysis to meet the cell increased energy needs [33]. The shift of tumor cell metabolic balance towards oxidative phosphorylation as a result of treatment is considered a favorable factor and is associated with transition to a less aggressive phenotype. With tumor oxidative status, increased oxygen consumption making the tumor more sensitive to hypoxia is observed along with decreased lactate production making tumor microenvironment more favorable for attack of effector T cells and natural killers, increased mitochondrial activity, nucleotide and lipid biosynthesis weakening, and decrease of tumor cells proliferative activity [37].

It should be noted that the YTwt wild-type NK cell line demonstrates no evident cytotoxic effect on glioblastoma cells unlike modified lines. Besides, FLIM data revealed α_*2*_ NAD(P)H value decrease, most likely associated with increased glycolysis and a more aggressive phenotype. One of the possible reasons of unmodified NK cells low cytotoxic activity established in our research may be the short co-culture time. So, the research of Heinrich et al. [38] showed the destruction of spheroids of the human glioblastoma U87MG line only after 5 days or more of incubation with NK cells.

Thus, the new YT–Vav1^+^CISH^–/–^ and YT–Vav1^+^B2M^–/–^ modified NK cell lines demonstrate greater cytotoxic activity against primary patient’s glioblastoma spheroids compared with the standard line, causing early metabolic rearrangements and evident decrease of tumor cells viability.

## Conclusion

Efficacy evaluation of new Vav1^+^CISH^-/–^ and YT– Vav1^+^B2M^–/–^ modified NK cell lines on a 3D glioblastoma tumor spheroids model via assessment of glioblastoma cells viability using fluorescent staining and of their metabolic status using the innovative FLIM method demonstrated the high cytotoxic activity of “enhanced” NK cell lines compared to the unmodified standard line. Early (after 2 h) metabolic rearrangements preceding cell death were shown of glioblastoma cells towards oxidative status caused by new modified lines effects. The obtained results are important to understand interaction mechanisms between glioblastoma and NK cells and the development of glioblastoma adoptive cell therapy.
